# Collaborating with cancer patients and informal caregivers in a European study on quality of life: protocol to embed patient and public involvement within the EUonQoL project

**DOI:** 10.1186/s40900-024-00597-9

**Published:** 2024-06-11

**Authors:** Merel Engelaar, Nanne Bos, Femke van Schelven, Nora Lorenzo i Sunyer, Norbert Couespel, Giovanni Apolone, Cinzia Brunelli, Augusto Caraceni, Montse Ferrer, Mogens Groenvold, Stein Kaasa, Gennaro Ciliberto, Claudio Lombardo, Ricardo Pietrobon, Gabriella Pravettoni, Aude Sirven, Hugo Vachon, Alexandra Gilbert, Jany Rademakers

**Affiliations:** 1 Netherlands Institute for Health Services Research (Nivel), Utrecht, The Netherlands; 2 European Cancer Organisation (ECO), Brussels, Belgium; 3 Fondazione IRCCS Istituto Nazionale Dei Tumori Di Milano, Milan, Italy; 4 Università Degli Studi Di Milano, Milan, Italy; 5 Hospital del Mar Research Institute, Barcelona, Spain; 6 Bispebjerg/Frederiksberg Hospital and University of Copenhagen, Copenhagen, Denmark; 7 Oslo Universitetssykehus HF, Oslo, Norway; 8 IRCCS National Cancer Institute Regina Elena, Rome, Italy; 9 Organisation of European Cancer Institutes, Brussels, Belgium; 10 SporeData OÜ, Tallinn, Estonia; 11 Istituto Europeo Di Oncologia IRCCS, Milan, Italy; 12 Unicancer, Paris, France; 13 Quality of Life Department, European Organisation for Research and Treatment of Cancer, Brussels, Belgium; 14 Leeds Institute for Medical Research, University of Leeds, St. James’s University Hospital, Leeds, UK; 15 Department of Family Medicine, Care and Public Health Research Institute (CAPHRI), Maastricht University, Maastricht, The Netherlands

**Keywords:** Patient and public involvement, Patient engagement, Patient participation, Co-researchers, Oncology

## Abstract

**Background:**

Patient and public involvement (PPI) has become an essential part of health research. There is a need for genuine involvement in order to ensure that research is relevant to patients. This can then improve the quality, relevance, and impact of health research, while at the same time reducing wasted research and in doing so bringing science and society closer together. Despite the increasing attention for this involvement, it is not yet common practice to report on proposed activities. An article reporting planned PPI could provide guidance and inspiration for the wider academic community in future activities. Therefore, this current article aims to describe the way in which PPI principles are incorporated in the research project called “Quality of Life in Oncology: measuring what matters for cancer patients and survivors in Europe (EUonQoL).” This project aims to develop a new set of questionnaires to enable cancer patients to assess their quality of life, entitled the EUonQoL-Kit.

**Methods:**

The first step is to recruit cancer patients and their informal caregivers as co-researchers in order to train them to collaborate with the researchers. Based on their skills and preferences, they are then assigned to several of the project’s work packages. Their individual roles, tasks, and responsibilities regarding the work packages, to which they have been assigned, are evaluated and adapted when necessary. The impact of their involvement is evaluated by both the researchers and co-researchers.

**Discussion:**

PPI is a complex and dynamic process. As such, the overall structure of the research may be defined while at the same time leaving room for certain aspects to be filled in later. Our research is, we believe, relevant as co-researcher involvement in such a large European project as EUonQoL is a new development.

## Abstract (plain language summary)

Patient and public involvement (PPI) is important in health research. This increases the relevance, quality, and impact of research, reduces wasted research, and promotes collaboration between science and society. There is growing attention for this involvement, but it is not yet common practice to report on planned PPI activities. This article addresses this gap by describing how its principles are integrated into the project called “Quality of Life in Oncology: measuring what matters for cancer patients and survivors in Europe (EUonQoL)”, a study that aims to develop a new set of quality of life questionnaires for cancer patients. The methods involve recruiting and training co-researchers who collaborate with the researchers. Co-researchers perform tasks based on their skills and preferences. Their involvement is constantly evaluated with both researchers and co-researchers and adjusted if necessary. Continuous support is provided, and evaluations are conducted to assess the process and impact of co-researcher involvement. PPI is a complex and dynamic process. Therefore, this article outlines the overall research structure while leaving room for later adjustments. The intention of publishing the proposed activities is to contribute insights into the research field and inform future delivery of PPI. Our PPI activities should provide a meaningful contribution to the research field as co-researcher involvement in such a large European project is new.

## Background

Patient and public involvement (PPI) has become an essential part of health research. It is defined as “research being carried out ‘with’ or ‘by’ members of the public rather than ‘to’, ‘about’ or ‘for’ them” [1]. It is increasingly recognized that those affected by research outcomes have skills and knowledge of equal importance to the researchers and therefore have a contribution to make [2]. Patients, it is argued, should have a right to inform research into their condition and that, reducing the knowledge gap between researchers and patients, is a moral duty [3, 4]. There is a need for the genuine involvement of patients in order to improve the quality, relevance, and impact of health research, while at the same time to reduce wastage of research and, in doing so, bring science and society closer together [5–8]. PPI can be applied at all stages of research, from clarifying a problem and formulating a question, to its implementation and dissemination. In this way it aims to improve the design and delivery of research in addition to providing data to answer the questions raised [9]. PPI means more than simply applying a certain method. Rather it is a critical attitude in which you come to a solution for a collectively defined problem through the involvement of those affected by it [10]. The important values and principles of PPI include respect, openness, inclusion, diversity, transparency, responsiveness, and accountability [11, 12].

There is increasing attention for both the reporting of PPI activities and the producing of guidance to help researchers plan and conduct it meaningfully. However, it is not often reported what the outcomes and impact of incorporating PPI principles into research have been [13]. Previous research showed that it is difficult to gain insight into the extent to which PPI contributes to the outcomes of a project as project teams find it hard to specify this [14]. As a result there is a lack of a strong evidence base for the impact of PPI in research [8, 15, 16]. Additionally, it is not yet common practice to report on proposed PPI activities and their expected impacts. As such an article reporting on planned PPI activities is important for transparency and may provide guidance and inspiration for the wider academic community on future activities [17]. PPI should offer many opportunities for patients and the public to learn skills which help both personally and professionally. Additionally, engaging in PPI is also a learning experience for researchers. This, we hope, will result in researchers being more prepared to engage in PPI in future research. And, it may too, give them more insight into the target population of their research thus strengthening the relevance of their results. Lastly, this should enhance the respect researchers share for the right of patients to have an input into the research that concerns them.

Here we describe the way in which PPI principles will be taken up by, and evaluated in, the research project called “Quality of Life in Oncology: measuring what matters for cancer patients and survivors in Europe” (EUonQoL, http://www.euonqol.eu/) [18]. A brief description of this project can be found in Table 1.

**Table 1:** Description of the EUonQoL project

The EUonQoL project aims to review existing quality of life scales in order to develop new metrics by harnessing the strengths, and overcoming the limitations of, previous tools. The EUonQoL-Kit, a new set of quality of life questionnaires designed for cancer patients in Europe, will be the product of this effort. It will form a new digital system for self-assessing the quality of one’s life, available in several European languages, and developed from the patient’s perspective [18]. The EUonQoL-Kit will be validated in a pilot study (Clinical Trials ID NCT05947903). Further description of the project and the participating organisations can be found on the EUonQoL website: http://www.euonqol.eu/	

## Methods

### The role of PPI within the EUonQoL project

**Fig. 1:**
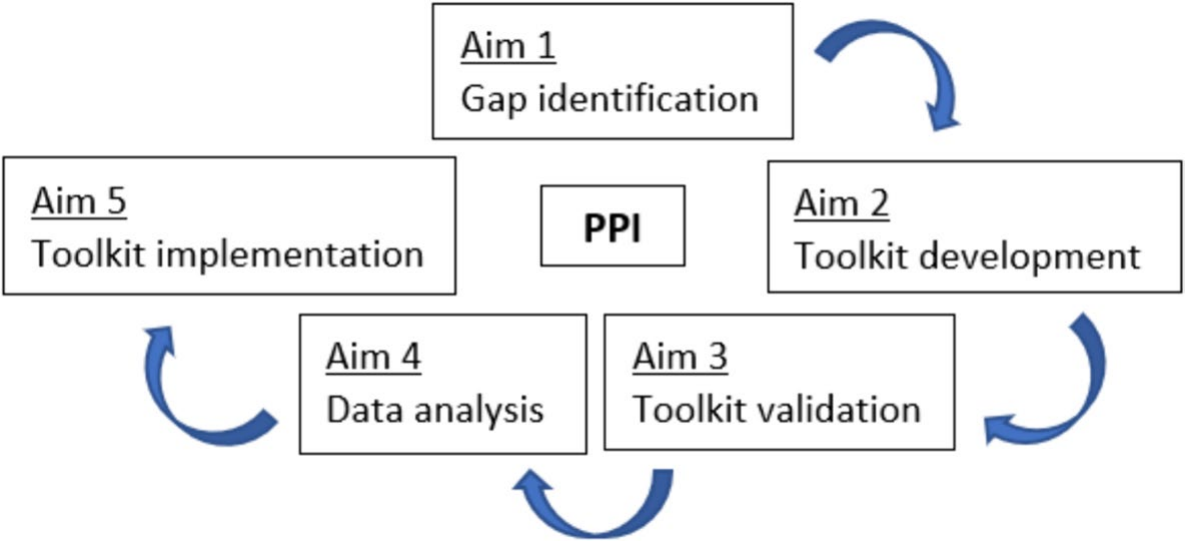
Toolkit development aims, adapted from Apolone & Brunelli [18]. PPI = Patient and Public Involvement

There are specific aims that need to be addressed in the development of the new EUonQoL-Kit (Fig. 1). Ideally, the core principle around which these aims revolve is PPI. The EUonQoL project is specifically based on these principles by involving cancer patients and their informal caregivers as co-researchers^1^ throughout. Within the different stages of the project, co-researchers can take on a variety of roles with different degrees of decision-making power.

### PPI handbook

The researchers responsible for facilitating PPI started by writing a handbook for the EUonQoL project on how to collaborate with co-researchers [19]. The involvement of patients in the development of the handbook was achieved by consulting members of the European Cancer Organisation’s Patient Advisory Committee.^2^ In order to safeguard co-researchers’ input into the handbook as it develops, it was declared a “living document” that will be updated annually with the latest insights and experiences. This will ensure that it recognizes the dynamic and iterative nature of PPI. The handbook is written specifically for the researchers involved in the EUonQoL project. However, in order to make it relevant for all researchers engaging in PPI, it has been published on the Nivel website. It aims to facilitate collaborations between researchers and co-researchers, taking PPI principles into account. The handbook contains a theoretical background on PPI and its benefits and barriers, followed by practical aspects that need to be considered when ensuring the involvement of patients, and good practices for collaborating with co-researchers. Finally, the handbook contains a checklist of items that have to be completed when research begins, such as an agreement on roles, tasks and responsibilities, language, frequency of contact, reimbursement, and support for co-researchers.

### Recruitment of co-researchers

Before launching the project it was estimated that six co-researchers would be required. Costs, however, were also a consideration. However, this number could change according to the participation and experiences of the co-researchers throughout the project. As a first step a varied group of co-researchers were recruited to the project to ensure a range of perspectives [11]. Therefore, extra attention was given during recruitment to diversity in age, gender, country of origin, cancer type, disease stage, and treatment phase. The criteria for the recruitment of co-researchers included: being eighteen years old or above; living in a European country; having experience of cancer as a patient or an informal caregiver; having a good command of English, in order to be able to communicate with the researchers; having the ability, equipment, and willingness to participate in digital meetings; and having the ability and willingness to travel to in-person meetings. Recruitment took place via a call for action that circulated on social media (LinkedIn, X) and through the newsletter of the Organisation of European Cancer Institutes. The potential co-researchers who expressed their interest, first received additional information via e-mail and were then invited for a video call to meet, in order to provide information about the project, and to discuss their potential involvement. Following these interviews, six co-researchers were selected by the researchers responsible for facilitating PPI in the project.

A system of reimbursement was set up in the EUonQoL project demonstrating the value placed on the expertise of co-researchers. They will receive financial compensation for the time they spend on the project. They will also be reimbursed for travel costs, and expenses related to the project.

### Training for co-researchers

Training then followed recruitment as co-researchers need specific skills to ensure that they can complete the tasks they agree to do [11]. A one-size-fits-all training approach is not useful for PPI projects. Instead, training formats must be developed together with the research team and the co-researchers [20, 21]. To be able to develop appropriate training, researchers were asked to describe the specific tasks they envisioned for the involvement of co-researchers in their work package during the research project. This overview of potential tasks was discussed with co-researchers, and training was developed for these specific tasks based on their needs, in addition to a basic training on the EUonQoL project. The training programme consisted of three sessions: an initial meeting, a second session where the project was discussed in-depth, and a third session, reserved for the specific training wishes of the co-researchers (Table 2).

**Table 2:** Content of the training program for co-researchers

Training session	Content/description
Training session 1 (initial meeting)	Introductions/getting to know each other
	Discussing the content, aims, and need for the research
	Formulating the ground rules for cooperation
	Exchanging personal contact details for communicating and reimbursement
Training session 2	Discussing research activities in more detail
	Aligning the proposed tasks with the co-researchers
	Discussing preferences in roles, tasks and responsibilities, and further training needs
Training session 3	A specific training session in the research techniques required such as Computerized Adaptive Testing
	Preparing co-researchers for the next steps including the practicalities of their involvement and the start of meetings to support them

### The assignment of co-researchers to their work packages

After training, the co-researchers were assigned to several of the project’s work packages, based on their skills and preferences. These tasks include, but are not limited to, the examples that are provided in Table 3. The specific individual roles, tasks, and responsibilities of co-researchers for their work packages are defined together with the researchers, evaluated continuously, and adapted when necessary, by using a tool called the Involvement Matrix [22]. This can be used by researchers and co-researchers to engage in regular dialogue about their ideas, needs and expectations in different phases and activities of their work package. The tool is visualised as a matrix, or mould, which describes five roles (Listener, Co-thinker, Advisor, Partner, and Decision-maker), and three stages (Preparation, Execution, and Implementation) in which co-researchers can participate. Ideally, the co-researchers take on roles with certain degrees of decision-making power (Advisor, Partner, Decision-maker). However, this depends on the preferences of co-researchers in which role they want to participate.

**Table 3:** Examples of co-researcher tasks within the EUonQoL project

Toolkit development aims	Examples of co-researcher tasks
Aim 1: Gap identification	Participation in meetings on work packages
	Interpretation of the results and outcomes of literature reviews
	Review of the reports and articles of literature reviews
Aim 2: Toolkit development	Participation in meetings on work packages
	Participation in consensus meetings, both online and in-person
	Providing input on the selection of quality of life measures
	Suggesting the language for novel items developed and for contextual wording within the toolkit
	Pre-test the draft toolkit prior to its usability testing
	Interpretation of the results from literature reviews related to clinical, socio-demographic, and psychological variables
	Providing input on the identification of domains and specification of indicators that link health care system factors to quality of life outcomes
Aim 3: Toolkit validation	Participation in meetings on work packages
	Providing input on Information Leaflet, Informed Consent and Data Protection forms for the pilot survey
	Help develop and review informative leaflets using layperson’s language and other dissemination material about the survey initiative
	Help develop and review standard operating procedures for data collection (i.e., how to identify and approach patients, how to train and/or assist them in the EUonQoL-Kit completion, how to perform data collection)
	Attend training workshops with health care professionals about standard operating procedures for data collection
	Help develop and review the dissemination of material, using lay language, about results from the survey
Aim 4: Data analysis	Participation in meetings on work packages
	Interpretation of the initial findings and providing input into the completion of the EUonQoL-Kit
Aim 5: Toolkit implementation	Participation in meetings on work packages
	Development and testing of the EUonQoL Implementation Guideline for the EUonQoL-Kit
	Providing feedback on the EUonQoL website
	Providing feedback on the EUonQoL Communication and Dissemination Plan content
	Disseminating study findings in their own network and engaging with a wider public
	Joint authorship of scientific and other publications resulting from EUonQoL project activities

Regular appointments between co-researchers and various types of researchers are planned during the EUonQoL project in order to ensure the best possible collaboration [20]. Collaboration between co-researchers and researchers from the different work packages takes place through participation in online and in-person meetings and also through consultation by other means, such as via e-mail. Additionally, support is provided to co-researchers on a regular basis by the researchers who are responsible for co-researchers’ involvement [20]. These meetings are organised every two weeks and aim to get to know each other better and to relate and share experiences of working as co-researchers. We also reflect on their roles and contributions in the teams in the various work packages and discuss specific topics of interest in more depth. Personal support may also be provided on request through bilateral conversations.

### The evaluation and reporting of PPI

To get a picture of the impact of PPI, it is important to evaluate and report appropriately on the context, process, and outcomes of PPI [23]. Therefore, in the EUonQoL project, we use the regular meetings with the co-researchers for continuous evaluations while at the same time making notes of these discussions. Additionally, we make notes about the input of co-researchers during meetings of the consortium in which major decisions are being made. At a later stage, these notes will be used to map the context and process of PPI within the project.

Researchers’ accounts of involvement provide a source of insight and learning as well. However, to date, these do not always describe, in sufficient detail, the context, mechanism, and expected outcome of the PPI approach [24]. Therefore, researchers and co-researchers are asked four times a year to fill in a digital evaluation form, in order to reflect on their collaboration and what benefits it brings. This is adapted from the existing PPI impact log [25]. If relevant, several researchers in the various work packages are invited for an informal conversation of about 30 min to help reflect, in more depth, on the participation, and also to guide any adaptations that need to be made in the collaboration.

Furthermore, systematic and standardised reporting of PPI approaches remains limited. To enhance future research using a PPI approach, it should be reported, precisely and self-consciously, how and at which level PPI was employed [26]. This will be guided in the EUonQoL project by using a tool called the Guidance for Reporting Involvement of Patients and the Public (GRIPP2) reporting checklist. GRIPP2 is used to improve the reporting of PPI in research [13]. Greater quality in reporting should gradually lead to the development of a stronger PPI evidence base that may help to bring together, better, different PPI studies [13].

### Dissemination

The results of the EUonQoL project will be disseminated through public reports, publications in peer-reviewed journals, presentations at relevant conferences, and more widely through patient organisations and general social, and other, media. It is essential that it is made clear what the co-researcher contributions to the project were [20]. This will be done by, for example, acknowledging their involvement on the project website and on social media, by joint authorship of publications – including peer-reviewed ones—and folders for patients and the public, and by presenting together at conferences.

### Ethical considerations

While ethical approval is not required for PPI, they can raise a number of ethical concerns. Care should be taken with the introduction of complex concepts, the use of challenging language, and the sensitive nature of some subjects [27]. In particular, the EUonQoL project is focussed on the assessment of health problems that co-researchers may have experienced or are still experiencing. In this case, the discussion of personal and sensitive experiences involved in PPI may be a burden. Researchers should make every effort to provide a safe environment where the individual can be heard and supported as needed.

We aim to incorporate the following good practices within each PPI activity:

- In general, researchers need to invest time and energy into establishing a good relationship with the co-researchers and in creating the right conditions for collaboration. This can be achieved by building up a bond of trust in order to be able to share ideas and give each other feedback [28]. This can also be achieved by avoiding power imbalances through rejecting an hierarchical approach [11, 29, 30], and making sure co-researchers are treated as equal partners who bring their own unique experiences and perspectives [9]. This should result in co-researchers being empowered and confident to engage with researchers and the project [23].
- Before collaboration starts between researchers and co-researchers, time should be set aside to discuss their reasons for involvement, and to share wishes, expectations, needs, and preferences regarding roles, tasks, and responsibilities [20, 28]. A tool that can be used to guide this conversation is the Involvement Matrix [22].
- Before a meeting or a specific research activity takes place, researchers should make sure to give co-researchers material to read in advance and ensure that the materials are sufficiently easy to understand [11].
- After a meeting or a specific research activity has taken place, the researchers should inform co-researchers about how their contributions will be used or not [20].

Additionally, a formal collaboration agreement was signed between the co-researchers and the research institute responsible for the involvement of co-researchers in the project.

## Discussion

This article aims to describe how PPI principles are incorporated into the EUonQoL project. It reported how co-researchers are recruited, trained, involved, and supported in the EUonQoL project. Additionally, it described how PPI activities are evaluated and reported.

It is not necessary to define every detail beforehand in order to give PPI meaning. PPI is a complex and dynamic process, which means that researchers can be presented with ideas and concepts they had not previously considered. Therefore, it is important for both researchers and co-researchers to be flexible and adapt to events, unexpected or otherwise, that might occur during the process, and to make decisions accordingly [27, 31]. Additionally, the specific areas of involvement have to fit with the co-researchers’ needs and preferences. Therefore, while the overall structure of the research should be described, there should, at the same time, be room left for certain aspects to be filled in later.

To our knowledge, co-researcher involvement in a European project as large as EUonQoL is a new development. Conducting research on such a large scale has consequences for how PPI principles can, and will be, embedded in the project. This also reflects in the requirements that were made of co-researchers during recruitment such as their ability to speak English, having digital skills, and being able to travel. Although unintended, these requirements contribute to an already existing underrepresentation in research of patients and the public who are in a more vulnerable position. This may include people with learning difficulties, the elderly, or people from minority ethnic groups [26]. Additionally, online meetings tend to have a more formal nature, making it harder to feel connected to each other and to the project [32]. The EUonQoL project involves a large consortium consisting of many parties and, therefore, significant project management skills are required to facilitate and support the involvement of co-researchers.

Co-researcher involvement in a large European project is a novelty. But, we believe that our PPI activities will provide a meaningful contribution to the research field. We aim to ensure the future delivery of PPI of a high quality by publishing our PPI proposals for this project. Identifying and sharing the differences that PPI makes to research can result in better research projects in the future [33]. It can demonstrate to the wider academic community the benefits of good PPI practice enabling them to identify opportunities to improve their own research in this field.

## Data Availability

No datasets were generated or analysed during the current study.

## Abbreviations

PPI: Patient and Public Involvement
EUonQoL: Quality of Life in Oncology: measuring what matters for cancer patients and survivors in Europe
GRIPP2: Guidance for Reporting Involvement of Patients and the Public

## Footnotes

1 In the EUonQoL project the term ‘co-researchers’ is used for people who have experienced cancer themselves and who now collaborate with the researchers.

2 The Patient Advisory Committee features representatives of 21 European patient advocacy groups and provides direct insight into the issues and challenges faced by current and former cancer patients and their relatives.
